# Plasmablastic Lymphoma Associated with Adjacent Mature Plasma Cell Population Exhibiting Opposite Light Chain Restriction

**DOI:** 10.1155/2020/8875547

**Published:** 2020-12-28

**Authors:** Karina Furlan, Ira Miller, Brett Mahon, Fernando A. Ocampo Gonzalez, Nicholas Ward

**Affiliations:** Department of Pathology, Rush University Medical Center, 1750 W Harrison St, 5th floor, Chicago, IL 60612, USA

## Abstract

Plasmablastic lymphoma (PBL) is an aggressive high-grade B cell lymphoma, considered a variant of diffuse large B cell lymphoma with approximately 75% mortality within 6-7 months. We describe an unusual case of PBL arising as a maxillary mass in an HIV-negative, nontransplanted 78-year-old female. Histologic examination revealed a diffuse infiltrate of anaplastic appearing cells exhibiting plasmablastic morphology with an adjacent contiguous infiltrate of mature appearing plasma cells. The PBL and mature plasma cell components both demonstrated an immunophenotype of CD20(-), CD38(+), and CD138(+). The two populations differed by the PBL featuring a high proliferation rate by Ki-67 (~95%) with coexpression of both c-MYC and EBV, while the mature plasma cell component featured a low proliferation rate by Ki-67 (~5%) without coexpression of c-MYC or EBV. Kappa/lambda staining demonstrated lambda light chain restriction involving the PBL, while the mature plasma cell infiltrate revealed kappa light chain restriction. Our findings describe the rare association of PBL with a synchronous distinct population of mature plasma cells exhibiting opposite light chain restriction.

## 1. Introduction

Plasmablastic lymphoma (PBL) was first described in 1997 as an aggressive high-grade B cell lymphoma with predilection of involving the oral cavity and strongly associated with HIV and EBV infection [1]. Histologic features are characterized by a diffuse proliferation of cells exhibiting immunoblastic morphology and immunophenotype suggestive of plasmacytic differentiation including positivity for CD38, CD138 (partial +), MUM1, BLIMP-1, and XBP-1, while generally negative for expression of B cell markers CD20 and PAX5 [2, 3]. EBV infection detected by EBV-encoded RNA (EBER) is positive in approximately 70% of cases. MYC protein expression is demonstrated in the majority of cases and correlates with MYC translocation or amplification [4]. It has since been further characterized by occurring not only in HIV-infected individuals but in other types of immunosuppressive states including posttransplant and elderly patients, most likely associated with immunosenescence [5]. Although originally described as occurring in the head and neck, particularly the oral cavity, other sites of involvement include the gastrointestinal tract, skin, lung, and less frequently lymph nodes [4–7]. The prognosis is typically regarded as poor, with the majority of patients having an overall survival of 6-7 months [5–7].

Here, we describe an HIV-negative, nontransplanted 78-year-old female diagnosed with a lambda light chain restricted PBL involving the maxillary sinus with a concurrent mature plasma cell population distributed predominately in a contiguous circumferential pattern around the PBL exhibiting kappa monoclonal light chain restriction. We investigated the significance of these two populations by morphologic, immunophenotypic, and molecular methods including next-generation sequencing (NGS) targeted gene panel (596 gene panel) and immunoglobulin heavy (IGH) chain clonality testing by NGS methods.

## 2. Case Report and Ancillary Studies

**Figure 1:**
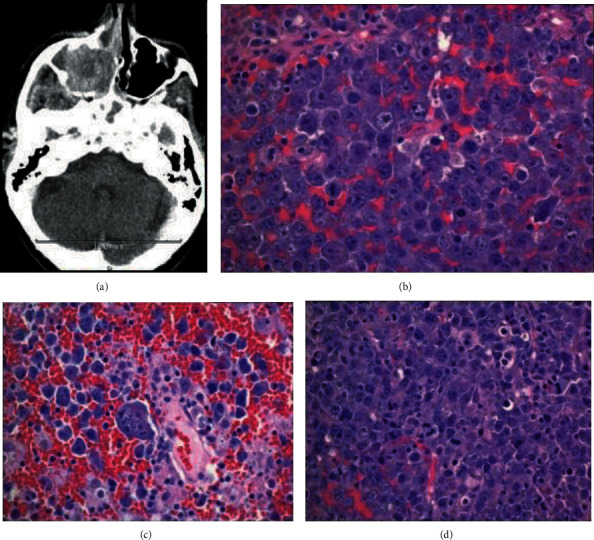
(a) CT right maxillary sinus mass. (b) Tumor cells forming sheets with a relatively cohesive growth pattern many exhibiting plasmablastic morphology (H&E, 400x). (c) Bizarre/anaplastic forms (H&E, 400x). (d) Abundant mitotic and apoptotic activity (H&E, 200x).

A 78-year-old female with no significant past medical history presented with chronic right-sided nasal obstruction with new-onset facial swelling. Computed tomography (CT) revealed a 3.3 cm mass centered in the right maxillary sinus extending into the right nasal cavity, right retro maxillary space, and along the inferior extraconal compartment of the right orbit (Figure 1(a)).

**Figure 2:**
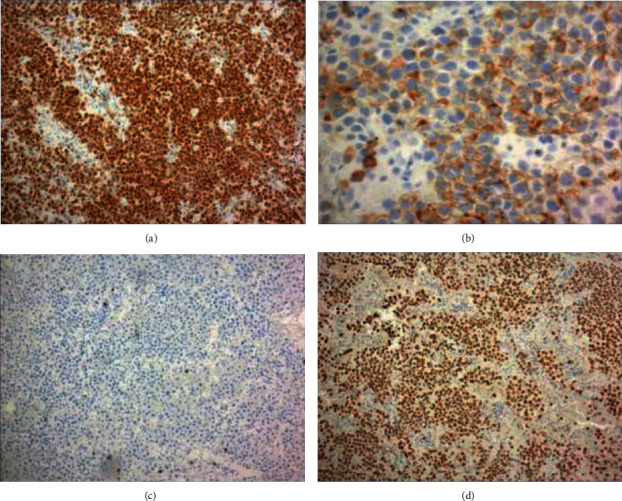
The large tumor cells are positive for MUM1 ((a) immunoperoxidase stain, 40x), partial positive for CD138 ((b) immunoperoxidase stain, 200x), and negative for CD20 ((c) immunoperoxidase stain, 40x) with Epstein–Barr virus-encoded RNA (EBER) transcripts identified by in situ hybridization ((d), 40x).

**Figure 3:**
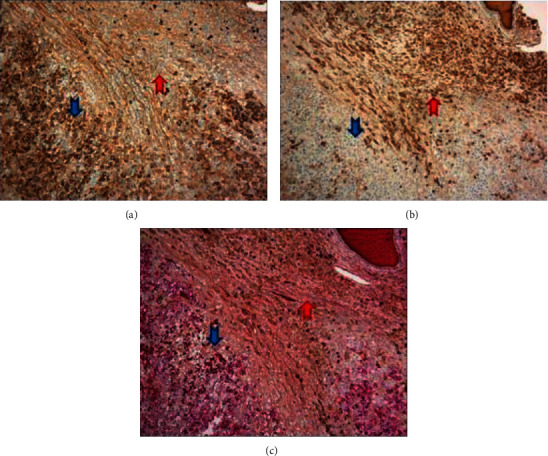
(a) Immunohistochemical stain for lambda, positive in the PBL (blue arrow) and predominately negative in the mature plasma cell component (red arrow) (100x). (b) Immunohistochemical stain for kappa, positive in the mature plasma cell component (red arrow) and essentially negative in the PBL cells (blue arrow) (100x). (c) Dual immunohistochemical stain for kappa and lambda showing lambda light chain restriction in the PBL cells (blue arrow) and kappa restriction in the mature plasma cell component (red arrow) (100x).

The patient underwent nasal endoscopy with bioptic material revealing effacement of architecture by a diffuse infiltrate composed primarily of large plasmablastic appearing cells with amphophilic cytoplasm, irregular nuclei, fine reticular chromatin, and occasional single, central nucleolus (Figures 1(b) and 1(c)). Brisk mitotic and apoptotic activity imparting a “starry sky” appearance and evidence of coagulative necrosis were noted (Figure 1(d)). Immunohistochemical profile (Figures 2(a) and 2(c)) was positive for CD138 (partial +) and MUM-1 (>30%), while negative for CD20. Epstein-Barr virus-encoded small RNA (EBER) was positive by in situ hybridization (ISH) (Figure 2(d)), c-MYC (>40%) had a strong and diffuse nuclear pattern of staining, and the proliferative rate by Ki-67 staining was high (>95%). Additional immunohistochemical staining revealed positivity for CD4, EMA, and PAX-5 (subset dim+), while AE1/AE3, CAM5.2, melan A, S100, CD117, myeloperoxidase, CD3, CD2, CD5, CD7, CD8, CD30, ALK-1, CD10, BCL-6, CD56, CD79a, CD34, TDT, and HHV-8 were negative. Kappa/lambda staining by in situ and immunohistochemical methods (Figures 3(a) and 3(b)) revealed that the plasmablastic tumor cells exhibited lambda restriction.

**Figure 4:**
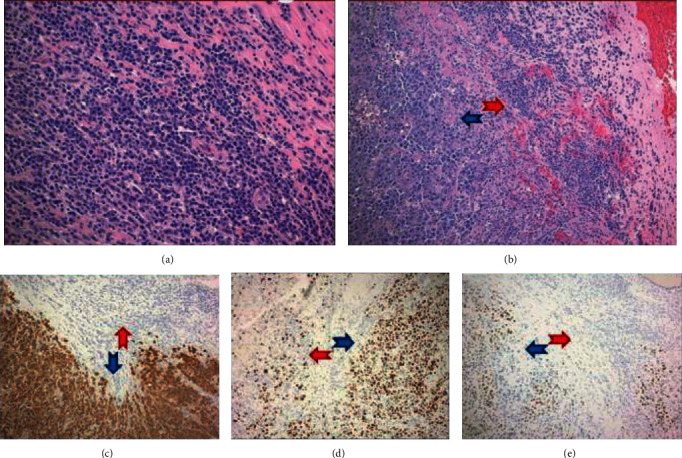
(a) The mature plasma cell population (H&E, 200x). (b) Interface of PBL (blue arrow) and mature plasma cell population (red arrow) (H&E, 100x). (c)–(e) Interface of PBL and mature plasma cell population showing opposite expression of EBER-ISH ((c), 40x), Ki-67 ((d), 40x), and c-MYC ((e), 40x).

A second distinct population of mature-appearing plasma cells without frank anaplastic/plasmablastic features was surrounding and contiguous to the PBL (Figures 4(a) and 4(b)). A careful morphologic examination found no notable admixed lymphocyte component/differentiation and no significant CD20 positivity, making a low-grade B cell lymphoma with marked plasmacytic differentiation unlikely. The immunophenotypic profile appeared distinct, as these cells showed negativity for EBER-ISH and lacked significant c-MYC expression, and the proliferation by Ki-67 staining was low (<5%) (Figures 4(c) and 4(e)). Kappa/lambda staining by both in situ and immunohistochemical methods demonstrated the mature plasma cells exhibited kappa light chain restriction (Figures 3(a) and 3(b)). A dual stain of kappa/lambda by immunohistochemical methods clearly highlights the respective light chain restricted populations (Figure 3(c)). FISH testing for MYC was negative for rearrangement, but three to five copies of the MYC gene were noted in 54% of cells analyzed. The final diagnosis was plasmablastic lymphoma with mention of an adjacent distinct mature appearing plasma cell population exhibiting opposite light chain restriction.

We subjected the two populations to further testing by mutation screening using a 596 gene panel and immunoglobulin heavy (IGH) chain clonality testing by next-generation sequencing (NGS) methods. The NGS targeted panel testing was performed after careful macrodissection, with DNA and RNA extracted from unstained formalin-fixed, paraffin-embedded sections. 100 nanograms (ng) of DNA for each tumor was mechanically sheared to an average size of 200 base pairs (bp) using a Covaris ultrasonicator. DNA libraries were prepared using the KAPA Hyper Prep Kit (Roche), hybridized to the xT probe set, and amplified with the KAPA HiFi HotStart ReadyMix (Roche). The amplified target-captured DNA tumor libraries were sequenced to an average unique on target depth of 500x on an Illumina HiSeq 4000. Samples were further assessed for uniformity with each sample required to have 95% of all targeted bp sequenced to a minimum depth of 300x. The NGS data for both the PBL and the light chain restricted mature appearing plasma cell population revealed no definitive pathologic variants, but multiple variants of unknown significance were noted (Tables 1(a) and 1(b)).

**Figure 5:**
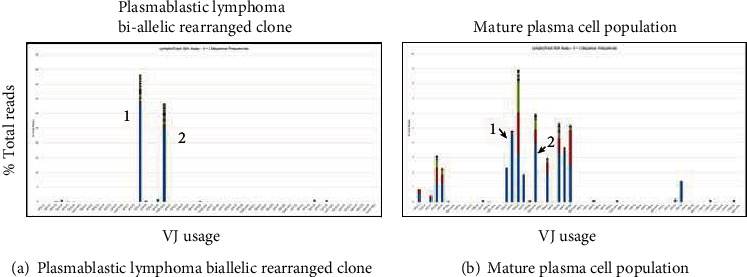
Immunoglobulin heavy chain clonality testing by next-generation sequencing. (a) Results from the plasmablastic lymphoma demonstrating two distinct peaks consistent with a biallelic rearranged clone. (b) Results from the mature plasma cell neoplasm with inclusive results due to a low number of reads, but demonstrating an oligoclonal pattern with low-level detection of the biallelic clone found in the PBL population.

Immunoglobulin heavy chain clonality testing by next-generation sequencing was performed in triplicate in both PBL and mature plasma cell populations after careful macrodissection. Clonality analysis was performed using the Lymphotrack IgH assay (Invivoscribe, CA). DNA was extracted from three separate regions of PBL and two distinct regions containing the mature plasma cell component from a single FFPE block. After PCR with FR1/2/3 and J primers, libraries were pooled and sequenced on a Miseq instrument according to the manufacturer's protocol. Fastq files were analyzed by the Lymphotrack software. A clonal read was defined if it was >2.5% of the total reads and was >10X the polyclonal background. Further characterization of the rearrangements was performed using the web-based IMGT/V-Quest and IgBLAST programs. Results of the PBL contained two clonal rearrangements with similar clonal frequencies. The FR1 primer set amplified both rearrangements much better than FR2 or FR3, but all three demonstrated similar findings. One rearrangement was productive (V3-20∗03/D3-22∗01/J3∗02), whereas the other was nonproductive (V3-71∗01/D3-16∗01,02/J5∗02) suggesting a malignant clone with biallelic rearrangements. The V regions of both rearrangements contained extensive somatic hypermutation, indicating a postgerminal center cell origin of the tumor. The three separate regions of PBL that were analyzed contained the same clonal rearrangements. The mature plasma cell component showed an oligoclonal pattern of rearrangements suggesting either paucity of plasma cells with reduced diversity in the regions analyzed or inability of FR1/FR2/FR3 primers to amplify rearrangements due to somatic hypermutation. In contrast to the adequate number of reads obtained with the FR1 primer set in the PBL, the mature plasma cell component did not amplify well with the FR1 primer set and generated a low number of reads. Clonal rearrangements were not identified in both mature plasma cell regions when the criteria of >2.5% read frequency and >10X the background were applied [8]. Nonetheless, the same two clonal rearrangements that were present in the plasmablastic lymphoma were detected in the mature plasma cell component, albeit at a low frequency. The FR1 results of this testing are shown in Figures 5(a) and 5(b).

**Figure 6:**
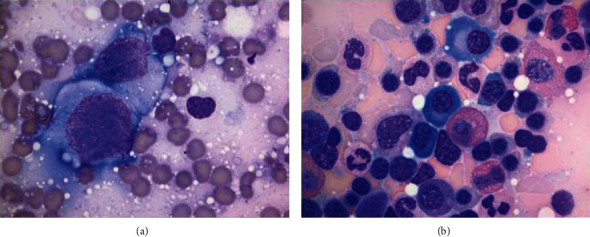
(a) Touch preparations from the PBL maxillary mass showing malignant cytology featuring enlarged nuclei, with high nuclear/cytoplasmic ratios, fine open reticular chromatin, and multiple prominent nucleoli (Diff-Quik stain, 1000x). (b) Aspirate smears from the bone marrow biopsy showing mature plasma cells (Wright-Giemsa, 1000x).

Subsequent bone marrow staging revealed trilineage hematopoiesis and was negative for involvement by plasmablastic lymphoma, but positive for a small plasma cell population (approximately 5-9% involvement) exhibiting kappa light chain restriction by both flow cytometric and in situ hybridization studies. No additional aberrant antigen expression by flow cytometry was detected. Morphology revealed mature plasma cells without frank anaplastic/plasmablastic features (Figures 6(a) and 6(b)). EBER-ISH and c-MYC were negative. Cytogenetic karyotype and FISH testing regarding MYC status were both normal without aberrations. Immunoglobulin clonality assay (IgH and IgK gene) using standardized BIOMED-2 multiplex PCR primers (InvivoScribe Technologies, San Diego CA, USA) [9, 10] was attempted and demonstrated no clonal bands when run by electrophoresis on polyacrylamide gels. However, given the low levels of involvement, a false negative result cannot be completely excluded.

Laboratory studies at the time of diagnosis revealed a serum protein electrophoresis (SPEP) with an elevated kappa/lambda ratio (9.0; reference range: 0.26–1.65) without definitive paraprotein (confirmed by immunofixation), with similar findings demonstrated in the urine. Complete blood count, calcium, liver function, and lactate dehydrogenase (LDH) studies were within the normal range. CT imaging demonstrated no masses or enlarged abdominal, pelvic, or thoracic lymph nodes. The patient ultimately opted to be treated by her local outside oncologist who reported that the patient was treated with 6 cycles of R-CHOP and was asymptomatic at a six-month follow-up. No further follow-up information is available in the electronic medical record.

## 3. Discussion

PBL is a rare and aggressive variant of diffuse large B cell lymphoma with the vast majority of cases associated with various states of immunodeficiency. A recent study by the Lysa group demonstrated in their cohort of 135 PBL cases that HIV-positive cases represented 42% of the cases, posttransplant 12%, and HIV-negative/nontransplanted 46% of the cases [5]. The HIV-negative/nontransplanted group was often associated with clinical findings of systemic inflammatory disease, history of cancer, and increased age, with only 5% of patients not showing any evidence of immune modulation or suppression. Interestingly, PBL patients with cooccurrence of HIV infection or in a posttransplant setting tend to show a significantly younger median age, 46 years and 55 years, respectively, when compared to those without these associations (71 years of age). In our case, a 78-year-old HIV-negative/nontransplanted patient without any other overt immune dysregulation, advanced age/immunosenescence appears likely attributable to the PBL, which is increasingly recognized in the literature [3, 8, 11].

The current case was associated with a unique finding of a concurrent distinct population of opposite light chain restricted mature plasma cells. Therefore, we sought to further understand the biology of these two populations by both a next-generation sequencing (NGS) targeted panel (596 gene panel) and immunoglobulin heavy (IGH) chain clonality testing by NGS methods. In the NGS targeted panel, both the PBL and the mature plasma cell population demonstrated among others a STAT3 missense mutation (c.1243G>A, p.E415K) at 38.9% and 14.6% variant allele frequencies, respectively. STAT3—typically implicated in T cell malignancy—is only occasionally reported in B cell-derived tumors. It is known to upregulate MYC protein expression and has also been implicated in the pathogenesis of ALK-positive large B cell lymphoma, another postgerminal center B cell malignancy that exhibits “plasmablastic” morphology [3, 12–14]. Of note, our case was ALK-negative by immunohistochemistry. The review of public databases of genetic variation (dbSNP and COSMIC) reveals the particular STAT3 mutation we observed; c.1243G>A (p.E415K) has only been reported as a somatic variant in a single liver carcinoma case (COSMIC database). Larger cohort studies need to be performed to establish the significance of this finding. The immunoglobulin heavy chain clonality analysis by NGS demonstrated the PBL contained two clonal rearrangements that featured extensive somatic hypermutation, consistent with postgerminal center origin tumor. Although the same two clonal rearrangements that were present in the PBL were detected in the mature plasma cell component, they did not meet the criteria, as mentioned previously to assign a clonal call. Contamination of the PBL at the time of macrodissection could presumably explain the low-level clonal rearrangements detected in the mature plasma cell population, particularly given the NGS panel findings of lower STAT3 and NF1 VAF mutations of uncertain significance in the mature plasma cell population. Additional testing for a kappa light chain (IgK) gene rearrangement was discussed, but this particular aspect of testing was not offered at the institution we pursued testing at.

PBL often features plasmacytic differentiation, which is enriched in cells with rounder nuclei, coarse chromatin, eccentric nuclei, and abundant basophilic cytoplasm with a paranuclear hof. These cases are found typically in the setting of nodal or nonoral involvement and are more likely in HIV-negative patients [1, 6, 15]. A key distinction of PBL exhibiting plasmacytic differentiation is the mature plasmacytic component retains the typical immunophenotype of a PBL including most cases showing EBV and MYC positivity with a high proliferation rate, findings not seen in our mature light chain restricted plasma cell population. An extensive review of the literature reveals only occasional case reports describing a similar finding of PBL with an associated distinct mature plasma cell population [16–18]. The clinical-pathological features of these cases are summarized in Table 2. Interestingly, given the aggressive nature of PBL, patients with plasmablastic tumors showing an associated mature plasma cell population appeared to respond well to therapy, although limited follow-up data is available.

We hypothesized that our case might represent a transformation of an underlying plasmacytoma into a PBL, possibly with the addition of MYC and EBV coinfection. As such, the mature plasma cell component possibly underwent a second round of extramedullary VJ recombination to express the lambda light chain at the same time that it gained copies of the MYC locus, and then subsequently underwent a germinal center type reaction to attain evident somatic hypermutation. An alternative hypothesis that is partially supported by the IgH heavy chain analysis by NGS suggests the mature plasma cells were potentially recruited in a reactive phenomenon that exhibits monotypic light chain restriction, yet are oligoclonal. Of note, the bone marrow findings revealed kappa monotypic plasma cells of the same light chain restriction noted in the mature plasma cell component of the maxillary mass. SPEP and urine protein electrophoresis both reported an elevated kappa/lambda ratio (9.0; reference range: 0.26–1.65), but without paraprotein.

Transformation to PBL has only been rarely described and typically attributed to a preexisting mature B cell neoplasm most often implicating chronic lymphocytic leukemia and follicular lymphoma [4]. Ambrosio et al. described a case of an oral cavity plasmacytoma with subsequent transformation to a clonally related PBL exhibiting the coexpression of EBV, MYC, and a high proliferation rate [17]. The true incidence of transformation or an associated mature plasma cell population is unknown and likely is underrecognized, as without rigorous examination for a background mature plasma cell population, many cases likely go unnoticed, particularly in a setting of limited tissue or core biopsy. Overall, PBL presumably represents a molecularly heterogeneous neoplasm deriving from postgerminal center B cells, yet the underlying pathogenesis remains incompletely understood particularly its association with mature plasma cell populations.

## Figures and Tables

**Table:** (a) Plasmablastic lymphoma

Gene symbol	Protein change	Allelic fraction
FANCC	c.1243G>A p.A415T missense variant	43.00%
STAT3	c.1243G>A p.E415K missense variant	38.90%
NF1	c.2278_2280del p.M760del Inframe deletion	38.50%
CD274 (PD-L1)	c.238A>G p.S80G missense variant	34.30%
KDM6A	c.1683+5_1683+6del splice region variant	20.10%
DOT1L	c.1905G>T p.R635S missense variant	18.40%
RB1	c.295T>G p.W99G missense variant	14.70%
SMARCE1	c.1079G>A p.G360D missense variant	13.90%
MAF	c.1023G>C p.R341S missense variant	7.60%
PAX3	c.499G>A p.E167K missense variant	6.10%

**Table:** (b) Mature plasma cell neoplasm

Gene symbol	Protein change	Allelic fraction
FANCC	c.1243G>A p.A415T missense variant	47.50%
STAT3	c.1243G>A p.E415K missense variant	14.60%
ETV1	c.593C>T p.P198L missense variant	9.90%
NF1	c.2278_2280del p.M760del Inframe deletion	9.50%
CIC	c.7517del p.P2506fs Frameshift	9.40%
PRSS1	c.32C>A p.A11E missense variant	7.50%

**Table 2:** Summary of cases published in the literature to date of plasmacytomas progressing to PBL.

Case	Age/sex	HIV/posttransplant	Location	Ki-67	c-MYC IHC	FISH (MYC)	Monoclonal IGHV	Bone marrow	Treatment	Survival
1 (Saito et al.)	34/M	(-/-)	Ileocecal with lymph node involvement	80-90%	Unknown	Unknown	Unknown	Negative	R-CHOP	7.5 years, still alive
2 (Ambrosio et al.)	74/M	Unknown	Maxillary sinus	95%	Positive	Negative	Positive	Negative	Complex (primarily CyBorD)	Disease free 11 months
3 (Qing et al.)	69/M	(-/-)	Generalized lymphadenopathy	95%	Unknown	Unknown	Unknown	Negative	CHOP	Alive; unclear on time frame
4 Current case	78/F	(-/-)	Maxillary sinus	95%	Positive	MYC amplification	Inconclusive	Mature appearing kappa restricted plasma cell population, no evidence of PBL	R-CHOP	Disease-free after 6 months

## Data Availability

Supporting documents can be requested from the first author (kcolossi@gmail.com). The data will be stored with the first author and senior author for 2 years.
